# Elimination of Pendimethalin in Integrated Rice and *Procambarus clarkii* Breeding Models and Dietary Risk Assessments

**DOI:** 10.3390/foods11091300

**Published:** 2022-04-29

**Authors:** Qiuhong Yang, Xiaohui Ai, Jing Dong, Yibin Yang, Shun Zhou, Yongtao Liu, Ning Xu

**Affiliations:** 1 Yangtze River Fisheries Research Institute, Chinese Academy of Fishery Sciences, Wuhan 430223, China; yangqh@yfi.ac.cn (Q.Y.); djing202211@163.com (J.D.); yangybin1985@163.com (Y.Y.); zhoushun19899@163.com (S.Z.); liuytao1979@163.com (Y.L.); ningxuxuning@163.com (N.X.); 2 Key Laboratory of Control of Quality and Safety for Aquatic Products, Ministry of Agriculture and Rural Affairs, Beijing 100141, China

**Keywords:** pendimethalin, *Procambarus clarkii*, elimination, residue, dietary risk, assessment

## Abstract

This study investigated elimination of the herbicide pendimethalin using an integrated rice and *Procambarus clarkii* breeding model of indoor and outdoor (pond culture) exposure tests. The pendimethalin levels in 484 samples from the primary rice and *P. clarkii* integrated breeding areas in Hubei province were monitored, and dietary risk assessments of pendimethalin were calculated. Pendimethalin was quantified using high-performance liquid chromatography tandem mass spectrometry, and detection levels were linear in the range of 1.0 to 10.0 μg/L, and peak areas were positively correlated with concentration, with a correlation coefficient of 0.9996. Recoveries ranged from 86.9 to 103.5%, and the limit of quantitation was 2.5 × 10^−4^ μg/L in water, and 1 × 10^−2^ μg/kg in tissues, sediments, and waterweeds. The dissipation rate of pendimethalin in tissues and water followed first-order kinetics, with half-lives of 0.51–5.64 d. In 484 samples taken from aquaculture farms, pendimethalin was detected in 8.67% of the samples at levels in the range of 1.95 to 8.26 μg/kg in Hubei province from 2018 to 2020. The maximum residue limit of pendimethalin in *P. clarkii* has not been established in China, but our dietary risk assessments indicated that consumption of *P. clarkii* from integrated rice farms was acceptable.

## 1. Introduction

The *Procambarus clarkii* (crayfish) was originally found in the southeastern United States, but was introduced into China in the late 1930s. With the rapid growth in the domestic market for *P. clarkii* and increasing economic benefits, its value has increased dramatically in recent years [1,2,3]. China has become the largest *P. clarkii* farming area in the world, accounting for more than 90% of the global production [4,5]. The model of “integrated rice and *P. clarkii* culture” is becoming popular globally due to its economic, social, and ecological benefits, which enables the agriculture and aquaculture industries to raise the utilization and productivity rates of rice paddies to the maximum extent [6,7,8,9]. The co-cultivation of rice with *P. clarkii* has steadily been increasing from its inception in 2017 from 8.5 to 15.86 million Chinese acres in just three years. Hubei province is the largest *P. clarkii* farming region, and its breeding area was approximately 7.9 million Chinese acres in 2019 [10,11,12]. The comprehensive co-cultivation reduces pesticide use, and there are now governmental technical specifications for cultivation and breeding [1]. However, control of pests and diseases will most likely result in an increase in pesticide use, and the entry of these chemicals into the breeding environment poses both risks and challenges for this food supply [13]. Therefore, it is necessary to collect relevant data of pesticide residuals and develop risk assessments for the quality and safety of *P. clarkii* in these environments.

Pendimethalin is an aniline herbicide developed by the American Cyanamide Company. Its primary mode of action is mitotic inhibition of broadleaf and grassy weeds at the meristems causing the plants to wither and die [14,15,16]. This herbicide is widely used in the field weeding of vegetables, cotton, soybeans, peanuts, corn, rice, and wheat, and can be used in a flexible manner. The effective period can be as long as 45–60 days, and can comprise the whole growth period of a crop [17]. The acute oral toxicity of pendimethalin is >5 g/kg b.wt. in humans (LD50 for rats >5000 g/kg b.wt.) [18]. It is of low acute toxicity to birds and bees, although it can adversely affect aquatic animals [19]. The U.S. Environmental Protection Agency (EPA) defines pendimethalin as a persistent, bio-accumulative, and toxic substance that has endocrine effects, and can form nitrosamines, and as such, it is classified as a possible human carcinogen (Grade C) [20,21,22]. Pendimethalin exposure can lead to an increase in the incidence of animal and human cancers, especially pancreatic, liver, and rectal cancer [23]. In a recent study, the cytotoxic potential of pendimethalin was deemed potentially genotoxic for the blue-spotted grouper and male rats [24,25]. This compound can also complex with DNA [26], and can function as an anti-androgen to interfere with endocrine function [27].

The United States has established the pendimethalin residue tolerance for lobster muscle tissue at 50 μg/kg [28]; in Japan, the limit standard for fish is 300 μg/kg [29]; and in the European Union, the maximum residue limits (MRLs) for aquatic pesticides are included in the new EU regulation on pesticide residue limit management. It contains 491 pesticides and 106 exempted pesticides. The MRLs stipulates that all pesticides other than those exempted by the regulation shall be subject to the limit of 10μg/kg [30]. Pendimethalin is a registered pesticide in China, but only for grains (MRL is 200 μg/kg for rice), oils (MRL is 100 μg/kg), and vegetables (MRL is 100–300 μg/kg), but not in aquatic products.

The current study utilized the *P. clarkii* to investigate the transfer of pendimethalin from water, sediment, and waterweed to *P. clarkii* muscle, hepatopancreas, and gill in integrated rice fields. We used indoor residue elimination and outdoor natural experiments, and measured pendimethalin levels in *P. clarkii* and in environmental samples using high-performance liquid chromatography to provide a theoretical basis for the use of the pesticide and its balance between efficacy and safety. Subsequently, we investigated the presence and concentration of pendimethalin herbicide residues in *P. clarkii* samples. Finally, the risk of exposure of Chinese people to the residues in *P. clarkii* was evaluated. This study provides valuable information for the safe consumption of *P. clarkii*.

## 2. Materials and Methods

### 2.1. Materials and Equipment

Ethyl acetate, methanol, and acetic acid were of chromatographic grade (CNW Resources, Napierville, Ill, USA), and 18.2 MΩ cm ultrapure water was produced using a Millipore filter system (Burlington, MA, USA). Neutral alumina column (1 g/3 mL), ethylenediamine-N-propylsilane (PSA) powder, and graphitized carbon black (GCB) powder were obtained from CNW. Pendimethalin standard (98.8%) was obtained from Dr. Ehrenstorfer (Augsburg, Germany) and pendimethalin EC (27%) from Jiangsu Longdeng Chemical (Jiangsu, China). The recommended application dosage was 0.27 mg/L.

Pendimethalin was quantified by HPLC linked to a Accela-TSQ Quantum Access Max triple quadrupole mass spectrometer equipped with Thermo LC-Quan 2.6 data acquisition and processing software (Thermo, Pittsburg, PA, USA); a Symmetry C18 column (100 mm × 2.1 mm, 3.5 μm) (Waters, Ireland) was applied to the LC system. The conditions of the chromatograph were as follows: mobile phase A was methanol containing 0.1% acetic acid, and mobile phase B was water containing 0.1% acetic acid; the flow rate was 0.3 mL min^−1^. The initial composition of the mobile phase was A: B 80:20 for 5 min, then 98:2 until 10 min, and a return to initial conditions. A 10 μL sample was injected into the column, and the column temperature was kept at 35 °C [31,32].

### 2.2. Pendimethalin Quantification

A pendimethalin working stock was prepared in methanol at 200 mg, and standard dilutions of 1, 2, 5, 10, and 20 µg/L were prepared in 80% methanol containing 0.1% acetic acid. A signal-to-noise ratio (S/N) = 3 was used to determine the limit of detection (LOD), and S/N = 10 was used to determine the limit of quantification (LOQ). Matrix effects (ME) were calculated by comparing the slope (A) of the matrix matching standard curve to the slope (B) of the solvent standard curve: ME = A /B × 100%. ME values of 0.8–1.2 indicated a weak ME, and ME < 0.8 was a strong effect, whereas ME > 1.2 was a matrix enhancement effect [33]. Recoveries were examined using pendimethalin spiked at 1×, 10×, and 100× LOQ.

### 2.3. Indoor Study Design

*P. clarkii* used in these experiments weighed, on average, 19.85 ± 7.62 g, and were purchased from Yumeikang Biotechnology (Yingcheng City, Hubei province, China). They were held for 1 week in a glass aquarium of 50 cm × 35 cm × 30 cm for 7 days in 20 individual boxes prior to experiments. The test water was fully aerated tap water, dissolved oxygen was maintained at 7.19 ± 0.24 mg/L, and the water temperature at 25.3 ± 1.2 °C. Water was changed and the animals were fed daily, and feeding was stopped 1 day before testing. Prior to experiments, animals from the bulk stock were randomly selected, and pendimethalin levels in their muscle, gill and hepatopancreas were determined using HPLC. Pendimethalin was not detected in any of these samples (data not shown).

The animals in triplicate tanks were exposed to pendimethalin at three concentrations according to GB/T 31270.21–2014 “Guidelines on environmental safety assessment for chemical pesticides”: a safe dose of 1.6 mg/L, a recommended dose of 0.27 mg/L, as well as controls lacking pesticide. The tissues were collected from six *P. clarkii* for each time point at 2, 4, 6, 8, and 12 h, and 1, 2, 3, 4, 5, 7, 14, 21, and 28 d. The rationale of tissue selection was based on the observations that the muscle is the major edible tissue; the hepatopancreas is the major metabolic and elimination organ; and the gill plays an important role in the absorption and excretion of xenobiotics in *P. clarkii* [34]. Tissues were homogenized in a high-speed food blender and stored at −20 °C prior to testing. Water samples (200 mL) were also collected in parallel and stored protected from light at 4 °C pending testing.

### 2.4. Outdoor Study Design

The outdoor simulation test was carried out in the integrated rice and *P. clarkii* cultivation project of the Yingcheng Branch of Yumeikang Biotechnology. The area of the test pond was about 1 mu (666.7 m^2^), and testing occurred in September where water temperature was 19–27 °C, pH was 7.24 ± 0.35, and dissolved oxygen was 7.22 ± 0.20 mg/L. Prior to testing, water, sediment, waterweeds, and *P. clarkii* were collected for pendimethalin quantification. The experimental ponds in the current experiments did not contain any detectable pendimethalin (not shown).

In triplicate ponds, pendimethalin EC was applied to the ponds as a spray at the recommended field dose at 1× and 0.5 × of the recommended dosage of 0.27 mg/L. Water, sediment, waterweeds, and *P. clarkii* samples were removed in triplicate for pendimethalin measurements at 2 and 12 h, and 1, 2, 3, 4, 5, 6, 7, 14, 21, and 28 d after pesticide application, and were stored at 4 °C and −20 °C.

### 2.5. Sampling and Testing of Rice and P. clarkii Growing Areas

Over the years 2018 to 2020, we also obtained 484 samples from the primary rice and *P. clarkii* integrated breeding areas in Qianjiang, Xiantao, Tianmen, Honghu, Gong’an, and Jianli districts in Hubei Province that included *P. clarkii*, water, sediment, waterweeds, rice, and animal feed.

### 2.6. Sample Pretreatment and Determination Method

Sample processing utilized the standard pretreatment and HPLC MS methods as previously reported [31,32].

Water samples (200 mL) were extracted by HLB cartridge and eluted with methylene chloride/acetone (1:1). The eluate was dried under a stream of nitrogen gas and adjusted to a final volume of 1 mL with methanol/water/acetic acid (80:20:0.1).

*P. clarkia* tissues, sediment, and waterweed samples (5 g each) were extracted twice by ethyl acetate/0.1% acetic acid. The neutral alumina column and the GCB sorbent (0.1 g) were used for purification. The samples were centrifuged, and 1 mL of the supernatant was used for analysis.

### 2.7. Data Processing

Standard curves, drug time curves, elimination equation, and half-life were calculated and drawn using Origin 7.0 (GraphPad, San Diego, CA, USA). The elimination equation adopted for the current study was C = C_0_ e^−kt^, where C represented the drug concentration, C_0_ the intercept of the residual elimination logarithmic curve (μg/kg), and k the elimination rate constant [35].

### 2.8. Dietary Intake Risk Assessment

Dietary intake risk assessments utilized the estimated maximum daily intake (EMDI) as previously reported [36]. EMDI was calculated according to Equation (1), and the risk quotient (RQ) d according to Equation (2):

EMDI = ∑ (MRL × F)(1)

RQ = 100% × EMDI/(ADI × bw)(2)

where EMDI, μg/d; MRL (maximum residue limit) refers to the maximum residue limit of pendimethalin in aquatic products, mg/kg; F is the estimated daily consumption of aquatic products, g/d; ADI (acceptable daily intake) is the allowable daily intake per kilogram of body weight, mg/(kg d); bw is the per capita body weight, kg.

RQ ≤ 100% indicates acceptable risk and a safe level; RQ > 100% indicates unacceptable risk.

## 3. Results

### 3.1. Pendimethalin Quantification

Pendimethalin quantification was performed using HPLC/MS, and the standard curve was linear in the range of 1.0–20.0 µg/L and could be represented by the equation Y = 6.59 × 10^5^ X − 2.0 × 10^5^ (r^2^ 0.9996). The fisher ration values confirmed the linearity of the standard curve, and the calculation process of F value can refer to reference [37] (Table 1 and Table 2).

The LOD and LOQ for pendimethalin in water were 1.0 × 10^−4^ μg/L and 2.5 × 10^−4^ μg/L, respectively. Pendimethalin levels in sediment, waterweeds, and *P. clarkii* tissues used as blank matrices that were spiked using pendimethalin stock solutions yielded a LOD of 5.0 × 10^−3^ μg/kg and an LOQ of 1.0 × 10^−2^ μg/kg for all matrices. We also examined matrix effects and ME for pendimethalin in water, waterweeds, and *P. clarkii* muscle and gill ranged from 0.8–1.2, indicating weak matrix effects. In contrast, the ME values of sediment and hepatopancreas were both > 1.2, indicating an enhancement effect, and for these samples, we used matrix-matched standard curves for quantification to ensure the accuracy of the data. Sample recoveries were examined using pendimethalin spikes at 1×, 10×, and 100× LOQ to blank water, sediment, waterweeds, and homogenized *P. clarkii* tissues (Table 3). The recoveries fell within an acceptable range for all matrices (86.9–103.5%, RSD 6.9–10.8%). This method, therefore, met the requirements for precise pendimethalin quantification, and the resulting LOQ and LOD for muscles, gills, hepatopancreas, sediments, and waterweeds were 5.0 × 10^−3^ μg/kg.

### 3.2. Indoor Residual Elimination Test Results

We examined the disposition of pendimethalin under indoor conditions in an enclosed water system to determine its accumulation within *P. clarkii* tissues at the safe concentration of 1.6 mg/L (Table 4) and the recommended dose of 0.27 mg/L (Table 5).

The *P. clarkii* exposed to 1.6 mg/L pendimethalin for only 2 h effectively and rapidly concentrated the chemical in the gills and hepatopancreas > 100-fold, but not in the muscle tissue. With increased exposure time, muscle levels peaked at 1 d and declined to undetectable levels by day 21. Pendimethalin levels in the gill were maximal at the initial sampling point and showed a biphasic distribution that decreased and reached a second maxima at 8 h and then steadily decreased, and at day 28, where the level was only 0.21 μg/kg. Strikingly, the hepatopancreas displayed a concentration of pendimethalin at day 1 of 10,462.65 μg/kg and decreased to about 0.3% of this level by day 28, although the levels were still elevated at ~30.95 μg/kg. The concentration of pendimethalin in the water was highest at the initial sampling point at 420.20 μg/kg, and then gradually decreased to undetectable levels by day 21 (Table 4).

The addition of pendimethalin at the recommended dose of 0.27 mg/L also revealed a time-dependent concentration effect in muscles, gills, and the hepatopancreas, although the initial levels were about 2-fold less at 2 h than were seen for the higher dose level. In general, the concentrations mirrored those of the higher dose, although the approach to the LOD values were reached more rapidly (Table 5).

**Figure 1:**
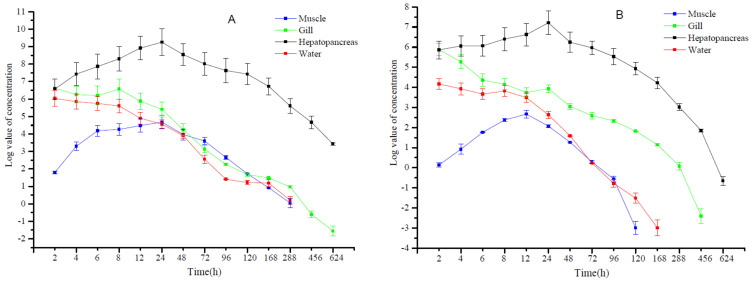
Log values of pendimethalin concentrations in the indicated tissues following addition to indoor tank water at (**A**) 1.6 and (**B**) 0.27 mg/L.

Pendimethalin was, therefore, eliminated from the tank water, and concentrated in *P. clarkii* tissues. The trend for tissue concentration indicated that muscle and gill tissues initially had accumulated the chemical, but then it was eliminated and generally followed the kinetics seen in the water. In contrast, the hepatopancreas concentrated the chemical and peaked at day 1 for both initial concentrations of the chemical. In all these cases, the chemical was not released back into the water from the animals (Figure 1).

### 3.3. Outdoor Simulation Test

We also examined these accumulation parameters in outdoor test ponds that had been sprayed with the recommended dosage of pendimethalin at 0.27 mg/L. The initial concentrations in the gill and hepatopancreas mirrored that of the indoor experiment, and were maximal at 2 h at 397.63 and 538.89 μg/kg, respectively, and decreased in gills, but accumulated in the hepatopancreas to a maximum of 1732.49 μg/kg at day 1. The trend for water and gill were similar, with a higher initial concentration that decreased in a constant manner over time. Muscles, sediments, and waterweeds all displayed a short-term increase and then a rapid decrease, with maximal levels on day 2. Interestingly, pendimethalin was eliminated from muscle tissues by day 21, but gill, hepatopancreas, water, sediment, and waterweed still retained levels ranging from 0.18 to 7.24 μg/kg on day 28 (Table 6).

Outdoor tests in ponds using 50% of the recommended dosage indicated that water levels showed a biphasic pattern where levels decreased from 29.59 to 9.15 μg/L by day 1 and rose to 16.84 μg/L on day 2, and then decreased gradually to a minimum of 0.78 μg/L at day 28. Pendimethalin in sediments decreased from a maximum at 12 h, and showed 2 other maxima at days 3 and 6, but high levels were still evident on day 28 at 3.64 μg/kg. Waterweeds also showed a concentration effect, with a maximum of 23.82 μg/kg on day 2 and 0.38 μg/kg on day 28. Similarly, tissues of *P. clarkii* displayed the most rapid initial accumulation (161.99 μg/kg) that rapidly decreased to undetectable levels by day 28. In muscle, accumulation peaked at day 2, but reached undetectable levels by day 7. In the hepatopancreas, the maximal accumulation occurred at day 1 (761.28 μg/kg), and then gradually decreased to 3.22 μg/kg by day 28 (Table 7).

**Figure 2:**
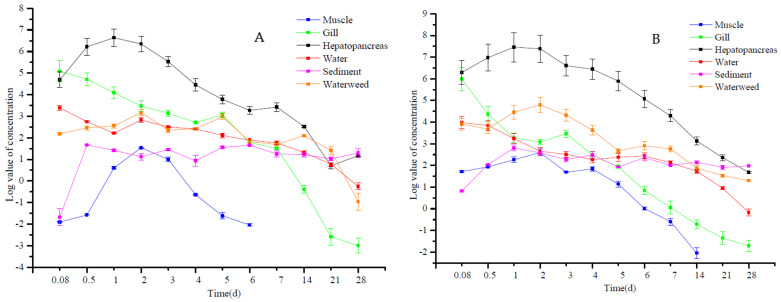
Log values for pendimethalin concentrations in the indicated tissues sprayed on outdoor test ponds at (**A**) 0.27 and (**B**) 0.14 mg/L.

The pond experiments demonstrated that pendimethalin in water and gills possessed the highest initial concentrations that then gradually decreased, whereas the levels in muscle, hepatopancreas, and waterweeds increased first and then decreased. The pendimethalin levels in the sediments fluctuated and, after day 7, plateaued and reached a stable level. The final concentrations in sediments were in direct proportion of the amount of the chemical applied to the ponds. In the other samples except for muscle, pendimethalin remained at detectable levels at day 28, and ranged from 0.18 to 7.24 and 0.05 to 3.64 for 1 × and 0.5 × the recommended applied dosage (Figure 2).

### 3.4. Comparison of Results between Indoor and Outdoor Simulation Test at the Same Dosage

The indoor and outdoor results data were processed using regression analysis elimination curve equations, correlation index (r^2^), and elimination half-life (T_1/2_) of pendimethalin concentration (C) at time (t). The indoor test resulted in T_1/2_ values for pendimethalin in muscle, gill, hepatopancreas, and water at 0.51, 3.17, 3.07, and 0.87 d, and 2.91, 4.50, 5.64, and 5.10 d for the outdoor test, respectively (Table 8).

We also performed a comprehensive study of 484 samples taken from rice and *P. clarkii* cultivation areas of Hubei Province. Overall, pendimethalin was detected in 71 (15%) samples. In *P. clarkii* muscle tissues and sediment, concentrations ranged from 3.59 to 6.84 μg/kg (21 samples), and 4.78 to 8.26 μg/kg (17 samples), respectively. Pendimethalin was not detected in water, waterweeds, or rice, whereas levels in feed ranged from 1.95–2.94 μg/kg, and were detected in 33 (19.05%) of the feed samples.

The national per capita consumption of aquatic products in China in 2019 was 13.6 kg [38], with an average daily consumption of 37.26 g. The National Food Safety Standard for pendimethalin ADI is 0.1 mg/ kg·bw. Using an average body weight of 65 kg, we therefore calculated the RQ value (Table 9). In our survey, concentrations of these random samples did not exceed 300 μg/kg, and presented an acceptable risk. In addition, the indoor and outdoor results of the current study indicated that pendimethalin in edible muscle tissue of *P. clarkii* was lower than this value, and was, therefore, an acceptable dietary risk.

## 4. Discussion

In this study, we found that pendimethalin accumulated in different tissues at different rates in *P. clarkii*. The chemical rapidly entered the gills and hepatopancreas, as was expected [39,40]. The liver and pancreas of crustaceans metabolize and eliminate drugs, but are also the main points of absorption [41]. For instance, 48 h exposure of imidacloprid and clothianidin to fish indicated that the liver and intestine had concentrated the chemical. Insecticidal amines are metabolized in the intestine and liver [42]. In experiments with *P. clarkii* exposed to florfenicol, the drug was primarily found in the liver and pancreas, but not the muscle [43]. Our results were consistent with these studies. Pendimethalin is a fat-soluble compound with an octanol-water partition coefficient of 5.18 [44], and thus, tends to accumulate in tissues with high fat content, such as the hepatopancreas; this was consistent with a previous study of herbicide residue accumulation in seafood [45]. Although pendimethalin in *P. clarkii* tended to accumulate in the hepatopancreas, muscle tissue could potentially be a storage tissue, and is a potential ecological and health risk.

Our indoor experiments yielded water levels of pendimethalin greater than for the pond study. Pond water is affected by environmental factors, such as temperature alterations, light, and microorganisms, and pendimethalin decomposed more rapidly in this environment. Nitrofural drugs in water are metabolized into semicarbazide under the effects of temperature and light, and malachite green levels in aquaculture waters are usually low due to the influence of sunlight [46,47]. We found that the T_1/2_ of pendimethalin in muscle, hepatopancreas, gills, and water were greater for the ponds, indicating an overall slower rate of degradation in the outdoor environment. A study of the elimination of minofloxacin in Chinese prawn culture systems indicated that sediment concentrations were 6–10 times that of the water, and thus, the drug had a tendency to transfer to sediment [48]. Soil organic matter affected the adsorption and degradation rate of promatogin, where higher organic matter levels led to higher adsorption [49]. Our results were consistent with these studies, and pendimethalin was retained at the highest levels in sediments. Pollutants, such as malachite green, also accumulate in sediments, and in general, the bottom mud is the final destination and carrier of most pollutants. The malachite green residues maintained a dynamic balance between water and sediment for an extended period, thereby causing secondary pollution to cultured fish [50]. A study of malachite green accumulation in the mud of 11 aquaculture farms in Northeast China found the chemical in only the 3 farms where it had been used in previous years [51]. Overall, these results indicated that the bottom mud not only adsorbs, but also maintains a dynamic balance with the aquaculture water. Our results were consistent with these reports. This also provides some ideas for the use of pendimethalin during *P. clarkii* breeding.

The current study also measured pendimethalin levels in production areas of rice *P. clarkii* farming in Hubei Province, but these samples may not fully reflect the pesticide pollution of the entire rice *P. clarkii* farming area. These regions can differ by season, breeding techniques, and pollution of pendimethalin and other pesticides. In the process of calculating the dietary risk quotient in this study, the maximum residue limit was 300 μg/kg, and the F value was based on the per capita consumption of aquatic products of residents, not the consumption of *P. clarkii* [52]. The consumption of *P. clarkii* in China accounted for 7.14% of the total national aquatic products in 2019, and 5.87% in 2018. Therefore, the risk quotient of *P. clarkii* may be lower. Nevertheless, the dietary risk assessment of the herbicide pendimethalin in *P. clarkii* has not been previously reported, and the results of this study can provide a reference for follow-up studies.

## Figures and Tables

**Table 1:** Six concentration (X) levels and their peak areas (Y) in triplicates.

X	Y			Y_ave_	Y_est_
0.5	196,928	166,928	178,433	180,763	203,237.5
1	548,270	528,270	543,943	540,161	535,960
2	1,099,811	118,731	104,245	440,929	1,201,405
5	3,374,365	2,904,365	3,043,453	3,107,394.33	3,197,740
10	6,431,819	6,231,819	6,234,131	6,299,256.33	6,524,965
20	13,191,544	13,041,544	12,893,432	13,042,173.3	13,179,415

Note: Y_ave_ is the average response at every concentration level. Y_est_ is the estimated response obtained by using Y = 6.59 × 10^5^; X − 2.0 × 10^5^.

**Table 2:** The calculation process of F value.

X	(Y-Y_ave_)^2^			(Y-Y_est_)^2^			(Yest-Y_ave_)^2^		
0.5	2.61 × 10^8^	1.91 × 10^8^	5.43 × 10^6^	3.98 × 10^7^	1.32 × 10^9^	6.15 × 10^8^	5.05 × 10^8^	5.05 × 10^8^	5.05 × 10^8^
1	6.58 × 10^7^	1.41 × 10^8^	1.43 × 10^7^	1.52 × 10^8^	5.91 × 10^7^	6.37 × 10^7^	1.76 × 10^7^	1.76 × 10^7^	1.76 × 10^7^
2	4.34 × 10^11^	1.04 × 10^11^	1.13 × 10^11^	1.03 × 10^10^	1.17 × 10^12^	1.20 × 10^12^	5.78 × 10^11^	5.78 × 10^11^	5.78 × 10^11^
5	7.13 × 10^10^	4.12 × 10^10^	4.09 × 10^9^	3.12 × 10^10^	8.61 × 10^10^	2.38 × 10^10^	8.16 × 10^9^	8.16 × 10^9^	8.16 × 10^9^
10	1.76 × 10^10^	4.55 × 10^9^	4.24 × 10^9^	8.68 × 10^9^	8.59 × 10^10^	8.46 × 10^10^	5.09 × 10^10^	5.09 × 10^10^	5.09 × 10^10^
20	2.23 × 10^10^	3.96 × 10^5^	2.21 × 10^10^	1.47 × 10^8^	1.90 × 10^10^	8.18 × 10^10^	1.88 × 10^10^	1.88 × 10^10^	1.88 × 10^10^
SS	SS_r_ = 8.39 × 10^11^			SS_ε_ = 2.81 × 10^12^			SS_lof_ = 1.97 × 10^12^		
DF	16			12			4		
SS/DF	δ_r_^2^ = 5.25 × 10^10^			δ_ε_^2^ = 2.81 × 10^12^			δ_lof_^2^ = 4.93 × 10^11^		
Fisher ratio	2.10								
Conclusion	2.10(calculated) < 3.259(tabulated at the 95% with 4 and 12 degrees of freedom), so the linearity of the standard curve is good and acceptable.								

Note: The residual error sum of squares (SS_r_), pure experimental error sum of squares (SS_ε_) and lack-of-fit error sum of squares (SS_lof_), degrees of freedom (DF), the critical value of F found in statistical tables (F_tabulated_), and Fisher ratio are calculated by δ_lof_^2^/δ_ε_^2^.

**Table 3:** Recoveries in different matrices with pendimethalin spikes at 1×, 10×, and 100 × LOQ.

Samples	Spike Level (μg kg^−1^)	Recovery1 (%)	Recovery2 (%)	Recovery3 (%)	Recovery4 (%)	Recovery5 (%)	Recovery6 (%)	Average Recovery (%)
Muscle	1 × LOQ	96.2	91.6	100.3	91.8	95.4	91.7	94.5
	10 × LOQ	87.3	86.4	96.4	106.3	89.6	90.5	92.8
	100 × LOQ	85.4	88.9	94.5	109.4	97.2	97.6	95.5
Gill	1 × LOQ	97.3	99.6	103.4	112.1	102.3	106.3	103.5
	10 × LOQ	110.2	96.3	85.3	94.6	89.6	102.6	96.4
	100 × LOQ	95.4	102.6	104.3	84.1	85.1	110.1	96.9
Hepatopancreas	1 × LOQ	86.2	110.1	96.5	106.9	97.7	103.8	100.2
	10 × LOQ	91.8	95.4	94.4	88.7	88.7	85.6	90.8
	100 × LOQ	87.7	86.3	82.9	94.2	90.3	105.8	91.2
Water	1 × LOQ	112.3	91.5	86.2	90.5	100.5	96.8	96.3
	10 × LOQ	94.4	86.8	89.5	101.4	98.6	86.4	92.9
	100 × LOQ	83.2	97.1	103.3	100.6	91.8	89.1	94.2
Sediment	1× LOQ	92.3	95.6	87.5	80.4	81.5	90.5	88
	10 × LOQ	106.2	85.6	93.4	99.6	87.1	101.4	95.6
	100 × LOQ	86.6	94.7	87.5	89.4	80.7	82.6	86.9
Waterweed	1 × LOQ	97.0	95.4	94.6	100.3	89.7	107.6	97.4
	10 × LOQ	95.2	86.3	82.9	86.6	94.6	91.7	89.6
	100 × LOQ	80.9	91.5	86.6	107.6	84.1	90.5	90.2

**Table 4:** Indoor exposure tests of pendimethalin added to tank water at 1.6 mg/L.

Time (h)	Muscle (μg/kg)	Gill (μg/kg)	Hepatopancreas (μg/kg)	Water (μg/L)
2	6.07 ± 0.47	746.57 ± 211.87	718.26 ± 326.35	420.20 ± 95.64
4	27.11 ± 10.62	518.85 ± 113.09	1691.60 ± 817.38	347.11 ± 79.38
6	65.89 ± 22.04	494.99 ± 204.13	2592.25 ± 1132.70	313.48 ± 62.05
8	70.94 ± 31.06	715.74 ± 328.42	4057.03 ± 950.86	271.11 ± 53.61
12	88.21 ± 41.23	355.94 ± 110.22	7516.75 ± 848.56	134.06 ± 29.75
24	108.01 ± 51.40	223.22 ± 76.05	10,462.65 ± 2148.21	95.41 ± 10.69
48	52.84 ± 20.65	69.75 ± 33.20	5141.91 ± 543.26	49.52 ± 4.53
72	36.48 ± 9.29	23.16 ± 8.63	3012.73 ± 664.06	12.88 ± 10.54
96	14.31 ± 3.10	9.60 ± 1.79	2062.32 ± 986.42	4.08 ± 1.12
120	5.63 ± 1.02	5.37 ± 2.35	1679.82 ± 421.63	3.38 ± 0.27
168	2.51 ± 1.03	4.43 ± 1.13	832.51 ± 129.05	3.28 ± 0.14
336	1.05 ± 0.06	2.66 ± 0.51	272.08 ± 62.23	1.23 ± 0.11
504	ND	0.55 ± 0.14	105.84 ± 36.31	ND
672	ND	0.21 ± 0.06	30.95 ± 2.05	ND

ND, not detectable.

**Table 5:** Indoor exposure tests of pendimethalin added to tank water at 0.27 mg/L.

Time (h)	Muscle (μg/kg)	Gill (μg/kg)	Hepatopancreas (μg/kg)	Water (μg/L)
2	1.15 ± 0.34	356.84 ± 20.22	352.12 ± 85.60	64.65 ± 13.65
4	2.25 ± 0.08	194.50 ± 32.64	429.66 ± 156.36	50.86 ± 20.68
6	5.83 ± 1.12	77.73 ± 23.69	432.20 ± 176.81	39.13 ± 14.53
8	10.76 ± 2.05	63.90 ± 15.66	603.95 ± 299.87	45.61 ± 15.61
12	14.40 ± 7.06	42.25 ± 9.38	750.79 ± 245.69	32.74 ± 10.87
24	7.95 ± 2.07	51.10 ± 7.12	1358.91 ± 354.28	13.93 ± 6.35
48	3.55 ± 1.04	21.05 ± 4.36	515.89 ± 157.91	4.87 ± 1.05
72	1.34 ± 0.55	13.36 ± 5.21	392.13 ± 29.68	1.26 ± 0.85
96	0.57 ± 0.23	10.29 ± 2.14	254.26 ± 56.94	0.46 ± 0.16
120	0.05 ± 0.04	6.18 ± 1.08	138.79 ± 23.42	0.22 ± 0.08
168	ND	3.15 ± 0.56	68.25 ± 16.95	0.05 ± 0.02
336	ND	1.08 ± 0.15	20.54 ± 5.63	ND
504	ND	0.09 ± 0.03	6.37 ± 2.17	ND
672	ND	ND	0.52 ± 0.11	ND

ND, not detectable.

**Table 6:** Pendimethalin quantification in test ponds sprayed with the recommended dose of 0.27 mg/L.

Time (d)	Muscle (μg/kg)	Gill (μg/kg)	Hepatopancreas (μg/kg)	Water (μg/L)	Sediment (μg/kg)	Waterweed (μg/kg)
0.083	5.59 ± 1.85	397.62 ± 225.35	538.89 ± 258.61	53.56 ± 15.63	2.28 ± 0.63	49.26 ± 12.84
0.5	6.90 ± 1.54	78.85 ± 30.22	1069.06 ± 446.42	46.75 ± 10.78	7.69 ± 1.21	39.85 ± 8.92
1	9.64 ± 3.48	26.14 ± 9.62	1732.49 ± 857.59	25.68 ± 9.14	16.54 ± 3.95	86.42 ± 24.31
2	13.42 ± 4.79	21.89 ± 3.05	1610.98 ± 538.32	14.21 ± 5.28	13.22 ± 1.68	120.46 ± 35.29
3	5.42 ± 1.35	32.13 ± 5.42	740.42 ± 111.64	12.33 ± 3.66	9.67 ± 2.45	75.06 ± 15.68
4	6.28 ± 2.86	12.04 ± 4.25	629.66 ± 106.32	9.65 ± 4.37	11.95 ± 5.14	38.08 ± 9.64
5	3.12 ± 0.27	6.96 ± 2.28	362.68 ± 83.73	10.82 ± 6.51	6.89 ± 1.22	14.38 ± 2.48
6	1.02 ± 0.53	2.35 ± 0.14	159.62 ± 53.02	11.49 ± 4.18	10.58 ± 3.74	18.30 ± 6.74
7	0.55 ± 0.21	1.06 ± 0.05	72.81 ± 15.97	8.37 ± 2.39	7.44 ± 0.85	15.88 ± 3.69
14	0.13 ± 0.08	0.49 ± 0.12	22.96 ± 6.64	5.64 ± 2.42	8.56 ± 1.86	6.56 ± 3.88
21	ND	0.26 ± 0.05	10.59 ± 3.96	2.59 ± 0.55	6.73 ± 2.64	4.62 ± 0.51
28	ND	0.18 ± 0.07	5.36 ± 0.64	0.84 ± 0.21	7.24 ± 0.95	3.68 ± 0.65

ND, not detectable.

**Table 7:** Pendimethalin quantification in test ponds sprayed with the 50% recommended dose (0.14 mg/L).

Time (d)	Muscle (μg/kg)	Gill (μg/kg)	Hepatopancreas (μg/kg)	Water (μg/L)	Sediment (μg/kg)	Waterweed (μg/kg)
0.083	0.15 ± 0.05	161.99 ± 125.36	109.06 ± 33.54	29.59 ± 3.28	0.19 ± 0.02	8.87 ± 1.57
0.5	0.21 ± 0.04	110.57 ± 20.22	500.98 ± 46.42	15.64 ± 1.29	5.31 ± 1.36	11.67 ± 2.56
1	1.83 ± 0.75	59.98 ± 15.62	761.28 ± 57.59	9.15 ± 0.84	4.14 ± 0.58	12.82 ± 2.47
2	4.67 ± 0.79	32.08 ± 13.05	571.79 ± 38.32	16.84 ± 2.84	3.07 ± 0.21	23.82 ± 3.68
3	2.72 ± 0.35	23.14 ± 4.42	252.09 ± 11.64	12.25 ± 1.43	4.28 ± 0.64	10.51 ± 2.61
4	0.53 ± 0.16	15.12 ± 1.25	85.88 ± 16.32	11.14 ± 0.77	2.55 ± 0.08	11.17 ± 1.79
5	0.20 ± 0.02	21.46 ± 3.28	43.68 ± 7.73	8.22 ± 3.14	4.74 ± 0.62	19.63 ± 3.82
6	0.13 ± 0.01	5.92 ± 0.73	26.40 ± 5.68	6.64 ± 1.05	5.26 ± 0.86	6.17 ± 0.46
7	ND	4.53 ± 1.64	30.66 ± 7.45	5.85 ± 2.16	3.51 ± 0.25	5.45 ± 0.69
14	ND	0.68 ± 0.16	12.46 ± 1.76	3.76 ± 0.56	3.39 ± 0.36	8.19 ± 1.39
21	ND	0.07 ± 0.02	2.04 ± 0.25	2.16 ± 0.43	2.79 ± 0.42	4.10 ± 0.14
28	ND	0.05 ± 0.03	3.22 ± 0.64	0.78 ± 0.21	3.64 ± 0.14	0.38 ± 0.02

ND, not detectable.

**Table 8:** Elimination equation, correlation index, and half-life of pendimethalin in *P. clarkii* tissues and water.

Group	Sampling Name	Elimination Equation	Correlation Index (r^2^)	Half-Life (T_1/2_)
Indoor exposure test	Muscle	C = 24.13 e^−1.08 t^	0.9457	0.51
	Gill	C = 59.57 e^−0.32 t^	0.9543	3.17
	Hepatopancreas	C = 799.12 e^−0.26 t^	0.9666	3.07
	Water	C = 50.95 e^−1.07 t^	0.9836	0.87
Outdoor natural culture test	Muscle	C = 18.52 e^−0.38 t^	0.8953	2.91
	Gill	C = 10.19 e^−0.36 t^	0.8752	4.50
	Hepatopancreas	C = 1055.40 e^−0.22 t^	0.8952	5.64
	Water	C = 26.76 e^−0.12 t^	0.8666	5.10

**Table 9:** The dietary risk of Chinese people according to MRL in different countries.

Country	Source	Consumption (g/d)	MRL (μg/kg)	RQ
Japan	Fish	37.26	300	0.172%
EU	Aquatic products	37.26	10	0.006%
USA	Lobster	37.26	50	0.029%
China	Rice	323.01	100	0.497%
China	Oils	26.03	200	0.080%
China	Vegetables	260.82	100–300	0.401–1.203%

Note: The maximum residue limit (MRL); the risk quotient (RQ).
